# Construction and Analysis of Hepatocellular Carcinoma Prognostic Model Based on Random Forest

**DOI:** 10.1155/2023/6707698

**Published:** 2023-01-12

**Authors:** Yikai Wang, Le Ma, Pengjun Xue, Bianni Qin, Ting Wang, Bo Li, Lina Wu, Liyan Zhao, Xiongtao Liu

**Affiliations:** ^1^Department of Infectious Diseases, The Second Affiliated Hospital of Xi'an Jiaotong University, Xi'an, Shaanxi 710004, China; ^2^Department of Operating Room, The Second Affiliated Hospital of Xi'an Jiaotong University, Xi'an 710004, Shaanxi Province, China

## Abstract

**Methods:**

Transcriptome data and clinical data of HCC were downloaded from the TCGA database. Screen important genes based on the random forest method, combined with differential expression genes (DEGs) to screen out important DEGs. The Kaplan‒Meier curve was used to evaluate its prognostic significance. Cox regression analysis was used to construct a survival prognosis prediction model, and the ROC curve was used to verify it. Finally, the mechanism of action was explored through GO and KEGG pathway enrichment and GeneMANIA coexpression analyses.

**Results:**

Seven important DEGs were identified, three were highly expressed and four were lowly expressed. Among them, GPRIN1, MYBL2, and GSTM5 were closely related to prognosis (*P* < 0.05). After the survival prognosis prediction model was established, the survival analysis showed that the survival time of the high-risk group was significantly shortened (*P* < 0.001), but the ROC analysis indicated that the model was not superior to staging. Twenty coexpressed genes were screened, and enrichment analysis indicated that glutathione metabolism was an important mechanism for these genes to regulate HCC progression.

**Conclusion:**

This study revealed the important DEGs affecting HCC progression and provided references for clinical assessment of patient prognosis and exploration of HCC progression mechanisms through the construction of predictive models and gene enrichment analysis.

## 1. Introduction

Liver cancer is the fifth most common cancer and the fourth leading cause of cancer-related death worldwide [1]. HCC is the most common type of liver cancer, accounting for about 75%–85%, and has the characteristics of a high mortality and high metastasis and recurrence rate [2]. Studies have shown that genetic mutations, chromosomal aberrations, molecular signaling pathways, and epigenetic dysregulation are all associated with the development of HCC [3]. At present, in addition to traditional surgical resection, radiofrequency ablation, transarterial chemotherapy, and other methods have been developed for the treatment of liver cancer [4]. Undeniably, surgical resection is still the most effective treatment for HCC. However, due to the insidious onset of HCC, many patients have already lost the opportunity for surgery when they come to the clinic. Even with surgical resection, the 5-year recurrence rate is as high as 70% [5], and the 5-year overall survival (OS) rate is only 15%–19% [6].

It is well known that the tumor microenvironment plays a crucial role in the occurrence and development of tumors. The interaction between various signaling molecules in the microenvironment is also a hot topic in tumor-related research. The occurrence of HCC is closely related to the inflammatory response of the environment, and 90% of HCC is associated with inflammation [7]. In the state of liver inflammation, the dysregulation of the interaction between cytokines, chemokines, and growth factors is an important cause of liver cancer [8, 9]. The original research on the IL-6, IL-1, and TGF-beta inflammatory molecules based on recent years for immune checkpoint research to further explore the development mechanism of HCC provided new insights. Studies have found that immune checkpoint molecules, such as programmed death-1(PD-1), cytotoxic T-lymphocyte antigen 4 (CTLA4), lymphocyte activating gene 3 protein (LAG-3), and mucin domain molecule 3 (TIM-3), are upregulated on liver cancer cells and tumor-specific T cells, which can lead to CD8^+^ T cell apoptosis and poor prognosis in patients [10]. At the same time, we cannot ignore that there are a large number of proangiogenic factors produced by cancer cells or tumor-infiltrating lymphocytes or macrophages in the tumor microenvironment, such as vascular endothelial growth factor (VEGF), which can promote tumor angiogenesis [11]. Angiogenesis is indispensable for tumor development, invasion, and metastasis [12]. On this basis, targeted drugs such as sorafenib, lenvatinib, VEGF inhibitors, and immune checkpoint inhibitors (ICIs) have significantly improved the prognosis of patients in recent years, but the overall treatment effect is still poor due to the changes in HCC heterogeneity and the continuous emergence of phenotypic drug resistance [13–19]. Therefore, it is particularly urgent to find a way to evaluate the disease early and take personalized treatment measures to improve the prognosis of patients.

The development of liver cancer is a multistep process caused by changes in signaling pathways triggered by multiple genes, and it shows high heterogeneity within tumors, between tumors, and between patients [20–24]. DEGs play an important role in this process. Therefore, considering the high heterogeneity of HCC, the limited treatment methods, and the poor prognosis of patients, it is more urgent to further explore the development mechanism of HCC and new survival and prognostic models. Nault et al. first identified a genetic marker associated with the development of HCC in 2013 [25]. Subsequent studies have also shown that gene mutations occurring in HCC can be used as biological markers for targeted therapies [26]. Although it has been demonstrated that programmed death ligand-1 (PD-L1) inhibitors combined with antiangiogenesis therapy can significantly improve the prognosis of patients with HCC [27], intervention-related toxicity and difficulty in determining the optimal dosing phase have hindered further benefit for patients [28, 29]. Therefore, the search for HCC-related dysfunctional genes is particularly important to elucidate the mechanisms underlying the development of the disease and to improve the prognosis of patients. Thanks to the rapid development of sequencing technology, many disease-related marker genes have been identified one after another, which has laid a solid foundation for the screening of HCC-related genes and the establishment of prognostic models. Public databases such as The Cancer Genome Atlas (TCGA) are useful tools to screen microarray data for DEGs associated with the development of HCC [30, 31].

In this study, we used a random forest algorithm to identify key genes expressed in HCC in the TCGA database and screened DEGs between HCC and normal samples. On this basis, 7 important DEGs were finally screened. Subsequently, we performed enrichment analysis on these 7 important DEGs and analyzed the expression levels of these genes in different clinical states. Furthermore, we performed survival analysis and COX regression analysis, constructed a prognostic risk score model, and plotted the receiver operating characteristic (ROC) curve. Finally, 20 coexpressed genes were screened by GeneMANIA, and GO and KEGG enrichment analyses were performed to further explore their biological functions and molecular pathways. The DEGs of HCC discovered in this study, as well as the constructed survival prognosis prediction model, are expected to provide new insights into the clinical treatment and biological mechanisms of HCC.

## 2. Materials and Methods

### 2.1. Data Source

The data for our study were extracted from The Cancer Genome Atlas (TCGA; https://portal.gdc.cancer.gov, up to July 31, 2022) database, which contains transcriptomic data of 374 HCC tumor samples and 50 normal samples, as well as 370 clinical samples and related data. We used data obtained from the TCGA database using Illumina HiSeq Systems, and the sequencing data format was a Counts file.

### 2.2. Random Forest Screening for Important Genes

Build a random forest model using the random forest package [32]. First, calculate the average model false positive rate based on out-of-band data for all genes and select 400 as the optimal number of trees to include in the random forest. Next, build a random forest model and use the Gini coefficient method to obtain dimension importance values for the random forest model. The genes with the top 30 importance values were selected for subsequent analysis.

### 2.3. Identification of DEGs

Expression data downloaded from TCGA were analyzed using the Limma package of R version 4.2.0 [33], and fold differential expression was calculated after removing or averaging probe sets without corresponding gene symbols or genes with multiple probe sets, respectively. The criteria for setting the DEG were as follows: genes with adjusted *P* value <0.05 and |logFC (fold change)| ≥ 1. Plot volcano plots using the ggplot2 package. Next, DEGs and genes screened by randomForest were combined to extract important differentially expressed genes.

### 2.4. Expression of DEGs in Different Clinical States

We further investigated the expression of DEGs and their association with different clinical states of HCC: event, age, gender, and stage. Violin plots were drawn using the ggplot2 package of the R software. Differences in gene expression among different groups were analyzed using SPSS 27.0. Definitions: ^*∗*^*P*  <  0.05, ^*∗∗*^*P*  <  0.01, ^*∗∗∗*^*P*  <  0.001.

### 2.5. Construction and Validation of an HCC Prognostic Risk Scoring Model

Kaplan‒Meier survival analysis was performed on the important DEGs using the R software survival and survminer packages, and the related survival curves were drawn.

Based on univariate and multivariate Cox regression analyses, a prognostic risk score model was constructed. According to the risk score grouping, prognostic analysis and Cox regression analysis were performed to verify whether the risk score could be used as an independent risk factor for evaluating the survival and prognosis of HCC patients. The specificity and sensitivity of the risk scoring model were verified using the R software pROC package, and the ROC was drawn using the ggplot2 package.

### 2.6. Enrichment Analyses of Important DEGs

Analysis of Gene Ontology (GO) and the Kyoto Encyclopedia of Genes and Genomes (KEGG) pathway enrichment plays a very important role in the annotation of gene products and the study of molecular mechanisms [34, 35]. We used enrichGO and enrichKEGG packages in the R language for enrichment, and adjusted *P* values <0.05 were considered significant; enrichment point plots were drawn using the ggplot2 package.

### 2.7. Analysis of the Relationship between DEGs Genes

We constructed a coexpression network of these genes using GeneMANIA (https://www.genemania.org/) [36] and identified associations within them. Subsequently, we further carried out enrichment analysis on the coexpressed genes of important DEGs, intending to explore their biological functions and molecular mechanisms.

## 3. Results

### 3.1. Identification of Important DEGs

**Figure 1:**
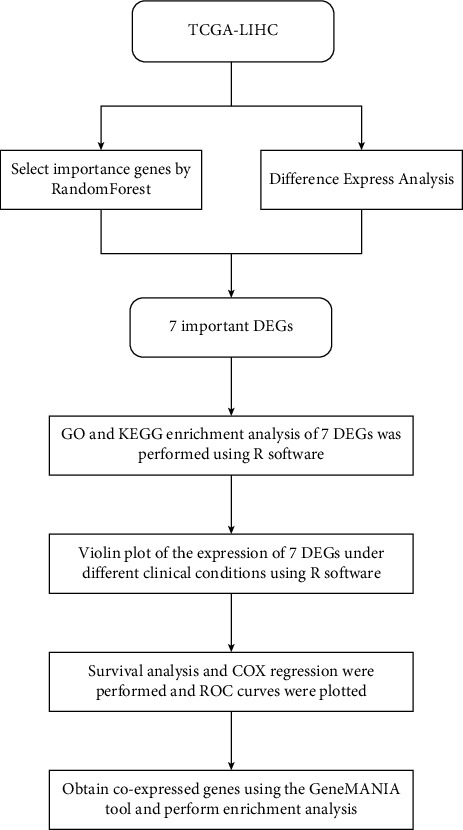
Flow chart of this study.

**Figure 2:**
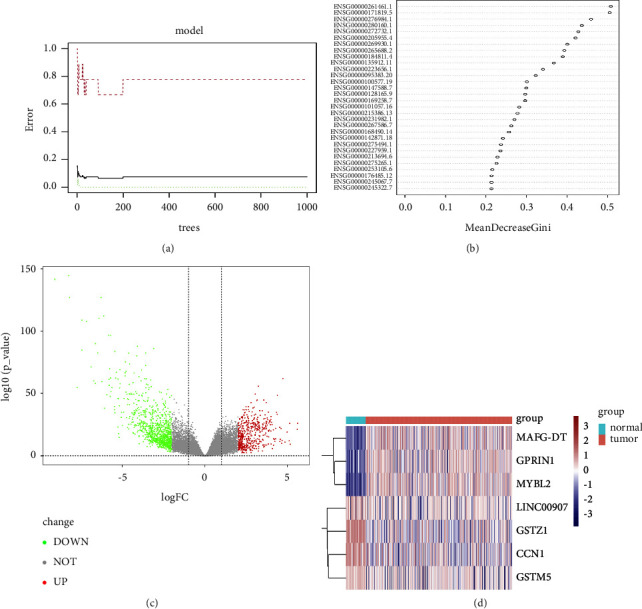
Identification of important DEGs: (a) the effect of the number of decision trees on the error rate. The *X*-axis represents the number of decision trees, and the *Y*-axis represents the error rate. When the number of decision trees is about 400, the error rate is relatively stable; (b) results of the Gini coefficient method in random forests. The *X*-axis represents genetic variables and the *Y*-axis represents the importance index; (c) the volcano diagram; (d) the heatmap diagram.

The research flowchart of this study is shown in Figure 1. First, we downloaded the expression profiling data of LIHC from the TCGA database along with clinical data. To find the genes with the greatest influence on the phenotype, we used the random forest method to screen. The relationship between the error of the reference model and the number of decision trees is shown in Figure 2(a). We selected 400 trees as the parameters of the final model, and the model error was stable at this time. We evaluated the final results using the Gini coefficient method and selected the top 30 genes as candidate genes (Table 1 and Figure 2(b)).

Next, we screened 1,564 DEGs using the Limma method and plotted the volcano (Figure 2(c)). After interacting with 30 candidate genes, 7 important DEGs were finally screened (Table 2). Analysis of the expression profiles of these seven DEGs revealed that MAFG-DT, GPRIN1, and MYBL2 were highly expressed in the tumor group, and LINC00907, GSTZ1, CCN1, and GSTM5 were lowly expressed in the tumor group (Figure 2(d)).

### 3.2. Expression and Clinical Parameters of Important DEGs in Patients

**Figure 3:**
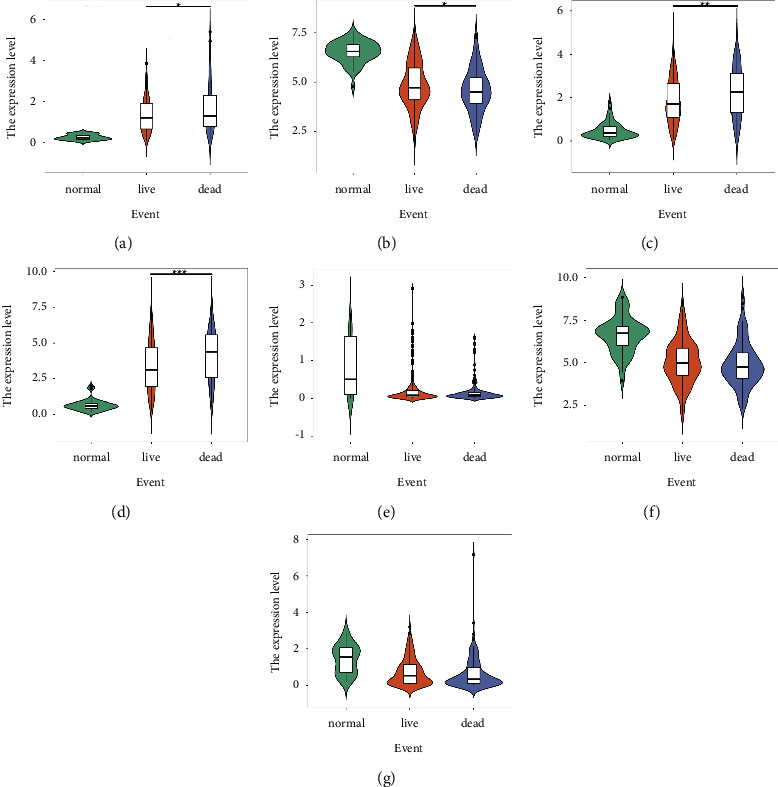
Expression of important DEGs in HCC patients with different events: (a) MAFG-DT; (b) GSTZ1; (c) GPRIN1; (d) MYBL2; (e) LINC00907; (f) CCN1; (g) GSTM5 (^^*∗*^^*P*  <  0.05, ^^*∗∗∗*^^*P*  <  0.001).

**Figure 4:**
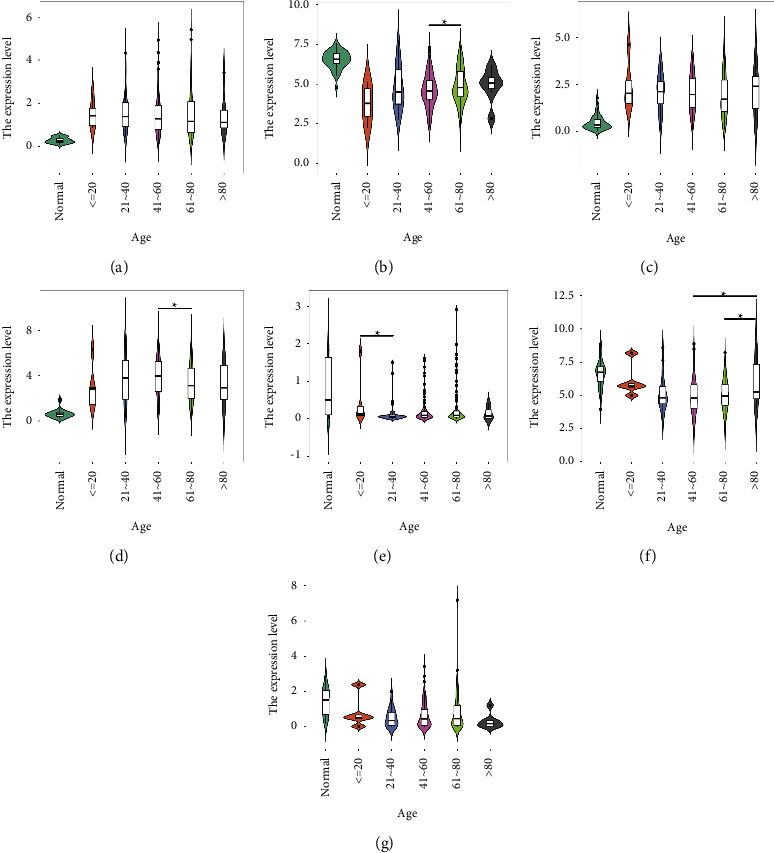
Expression of important DEGs in HCC patients with different ages: (a) MAFG-DT; (b) GSTZ1; (c) GPRIN1; (d) MYBL2; (e) LINC00907; (f) CCN1; (g) GSTM5 (^^*∗*^^*P*  <  0.05).

**Figure 5:**
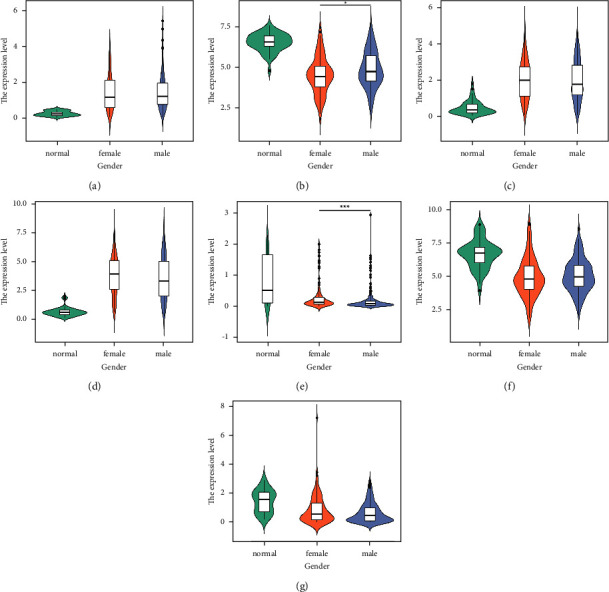
Expression of important DEGs in HCC patients with different genders: (a) MAFG-DT; (b) GSTZ1; (c) GPRIN1; (d) MYBL2; (e) LINC00907; (f) CCN1; (g) GSTM5 (^^*∗*^^*P*  <  0.05, ^^*∗∗∗*^^*P*  <  0.001).

**Figure 6:**
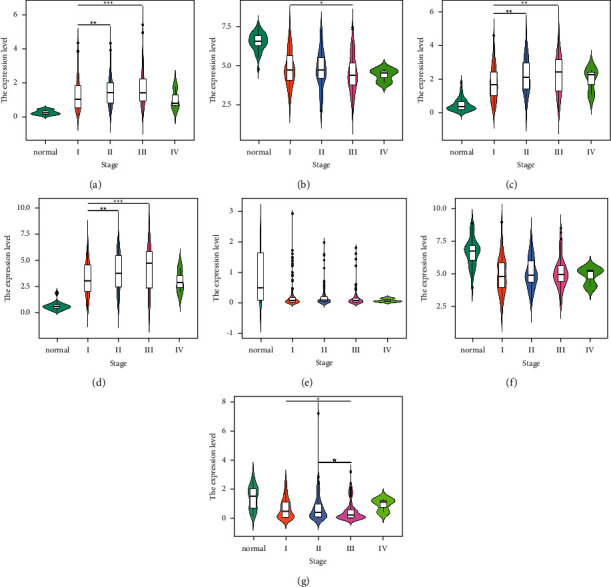
Expression of important DEGs in HCC patients with different stages: (a) MAFG-DT; (b) GSTZ1; (c) GPRIN1; (d) MYBL2; (e) LINC00907; (f) CCN1; (g) GSTM5 (^^*∗*^^*P*  <  0.05, ^^*∗∗*^^*P*  <  0.01, ^^*∗∗∗*^^*P*  <  0.001).

To further analyze the relationship between these seven important DEGs and clinical status, we used SPSS to compare the expression differences of each gene under different groups, and used the ggplot2 package of R software to draw a violin plot (Figures 3–6).

According to the outcome of patience, we divided the patients into the normal group, live group, and dead group. The results showed that compared with the normal group, the live and death groups showed significant differences in each gene (*P*  <  0.001). In addition, GSTZ1 expression was significantly higher in the live group than that in the death group (*P*=0.042489), while MAFG (*P*=0.047899), GPRIN1 (*P*=0.003478), and MYBL2 (*P*=0.000164) were significantly lower (Figure 3).

Stratified analysis by age revealed that GSTZ1 expression was significantly lower in the 41–60 years group than that in the 61–80 years group (*P*=0.037252), while the opposite was true for MYBL2 (*P*=0.017321). The expression of LINC00907 in the ≤20 years group was significantly higher than that in the 21–40 years group (*P*=0.035828). In addition, the expression of CCN1 was significantly lower in the group of 41–60 years (*P*=0.03869) and 61–80 years (*P*=0.026584) than in that of the over 80-year-old group. There was no significant difference in the expression of the remaining genes among the age groups (*P*  >  0.05) (Figure 4).

By analyzing the expression levels of each gene in patients of different genders, we found that the expression level of GSTZ1 (*P*=0.010058) in female patients was significantly lower than that in male patients, while LINC00907 (*P*=0.000144) was just the opposite (Figure 5).

The expression level of MAFG-DT was significantly lower in stage I than in stages II (*P*=0.006818) and III (*P*=0.000635) when grouped according to the HCC stage. However, the expression level of GSTZ1 in stage I was significantly higher than that in stage III (*P*=0.041631). In addition, GPRIN1 expression was significantly lower in stage I compared with stage II (*P*=0.001831) and stage III (*P*=0.001096), and the same difference was observed with MYBL2 (*P*=0.009085;  *P*=0.000282). In contrast, the expression level of GSTM5 in stage III was significantly lower than that in stages I (*P*=0.032924) and II (*P*=0.030159) (Figure 6).

### 3.3. Impact of Important DEGs on Patients' OS

**Figure 7:**
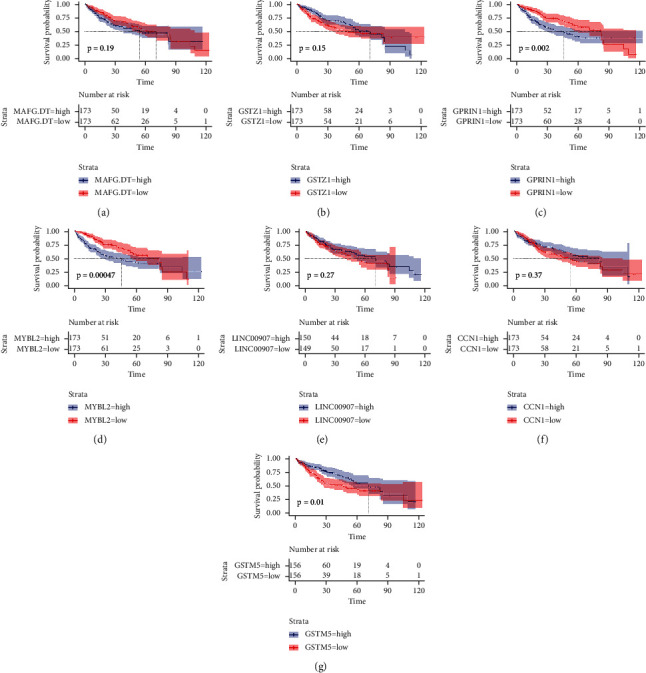
Survival curves of the relationship between common DEGs and prognosis of HCC patients: (a) MAFG-DT; (b) GSTZ1; (c) GPRIN1; (d) MYBL2; (e) LINC00907; (f) CCN1; (g) GSTM5.

We performed survival analysis using the survival and survminer packages of R software and used survival curves combined with log-rank tests to assess the impact of important DEGs on patient OS. As shown in Figure 7, high expression of GPRIN1 and MYBL2 indicated better prognosis of patients (*P*=0.002; *P*=0.00047), while patients with low expression of GSTM5 had better prognosis (*P*=0.01). However, MAFG-DT, GSTZ1, LINC00907, and CCN1 were not associated with patient prognosis (all *P*  >  0.05).

### 3.4. Construction and Validation of a Prognostic Risk Model for HCC Patients

**Figure 8:**
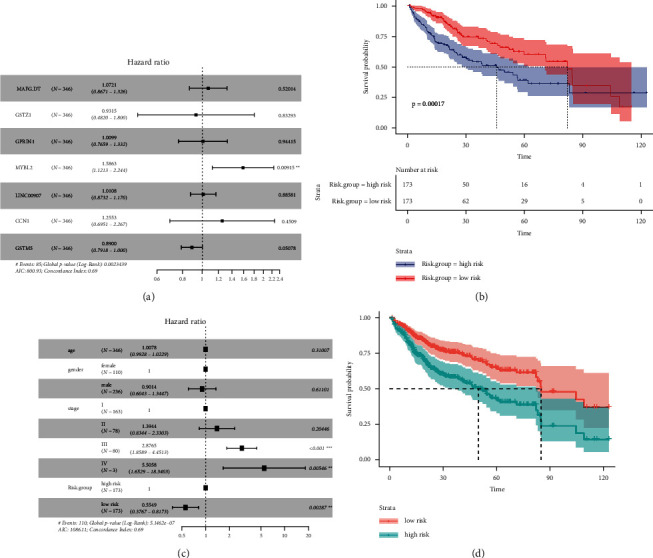
Prognostic analysis of HCC patients: (a) multivariate Cox analysis of important DEGs for the prognosis of HCC patients; (b) survival analysis of high-risk and low-risk groups; (c) multivariate Cox analysis of the prognosis of HCC patients with different clinical status; (d) Cox proportional-hazards model (^^*∗*^^*P*  <  0.05, ^^*∗∗*^^*P*  <  0.01, ^^*∗∗∗*^^*P*  <  0.001).

First, the selected seven important DEGs were combined with survival time and survival status and then included in a multivariate Cox regression analysis (Table 3 and Figure 8(a)), and a prognostic risk score model was constructed based on the results. The risk score calculation method is 7 important as the product of DEGs expression level and risk coefficient. The specific risk score model is risk score = “MAFG-DT”*∗* 0.069645 − “GSTZ1” *∗* 0.070909 + “GPRIN1” *∗* 0.009885 + “MYBL2” *∗* 0.461418 + “LINC00907” *∗* 0.010721 + “CCN1” *∗* 0.227363 − “GSTM5” *∗* 0.116514.

We divided 346 HCC samples into a high-risk group and a low-risk group, with 173 cases in each group, using the median risk value (2.4211) as the cutoff value. Survival analysis showed that there was a significant difference between the two groups (*P*=0.00017), and the survival time of the low-risk group was significantly longer than that of the high-risk group (Figure 8(b)).

The results of univariate Cox regression analysis showed that the risk score model was significantly correlated with survival time and survival status (hazard ratio = 0.5066, 95% Confidence interval = 0.353–0.727, *P*=0.000224); further multivariate Cox regression analysis results also showed that the risk score model was closely related to the prognosis status (hazard ratio = 0.5549, 95% confidence interval = 0.3767–0.8173, *P*=0.00287) (Table 4 and Figure 8(c)). The subsequent proportional hazard analysis also confirmed that patients with a lower risk score had a better prognosis (Figure 8(d)).

**Figure 9:**
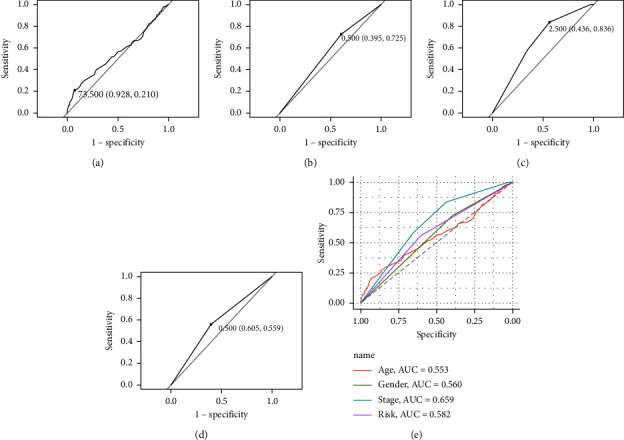
Clinical characteristics and model prediction of survival ROC curve: (a) age; (b) gender; (c) stage; (d) risk group; (e) all characteristics and AUC.

Finally, using ROC to evaluate the relationship between clinical characteristics and the prognosis of HCC patients, the results showed that the risk scoring model (AUC = 0.582) was the second most important factor for prognosis after stage (AUC = 0.659) (Figure 9).

### 3.5. Enrichment Analysis and Interaction Analysis

**Figure 10:**
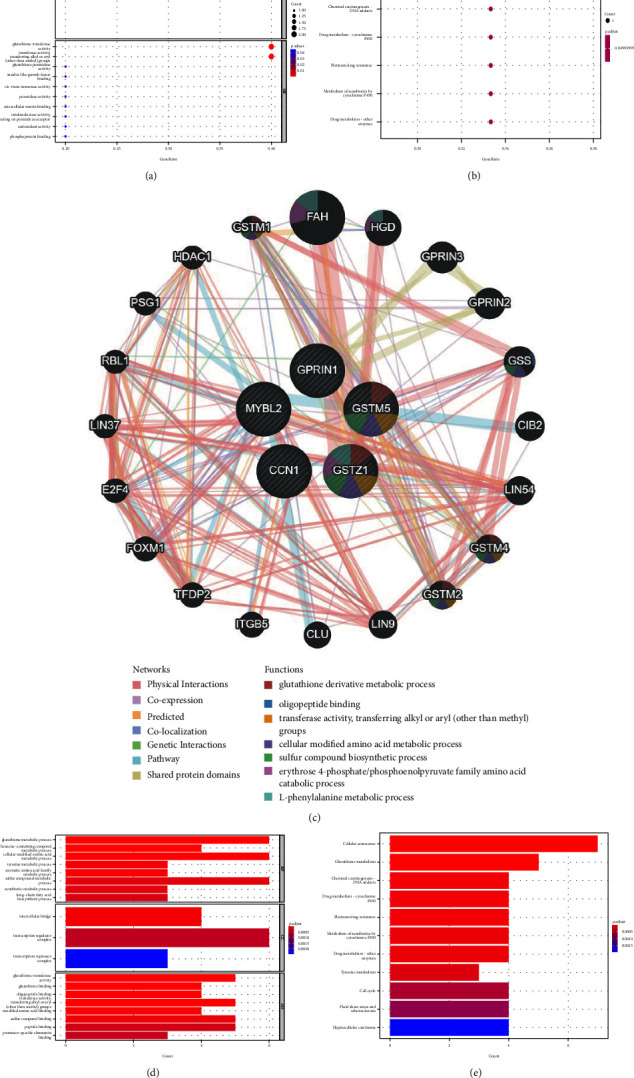
Enrichment analysis and coexpression analysis of important DEGs: (a) GO enrichment analysis of 7 important DEGs; (b) KEGG enrichment analysis of 7 important DEGs; (c) analysis of important DEGs and their interacting genes by GeneMANIA; (d) GO enrichment analysis of 25 interacting genes; (e) KEGG enrichment analysis of 25 interacting genes.

To further explore the mechanism of action and signaling pathway of important DEGs, we performed GO and KEGG enrichment analyses on them. GO enrichment analysis found that important DEGs were mainly enriched in the glutathione metabolic process (*P*=0.000117), glutathione transferase activity (*P*=1.91*E* − 05), and transferase activity, transferring alkyl or aryl (other than methyl) groups (*P*=0.0001) (Table 5 and Figure 10(a)). KEGG enrichment analysis showed that important DEGs were mainly enriched in the following seven pathways: tyrosine metabolism (*P*=0.01318), glutathione metabolism (*P*=0.020815), chemical carcinogenesis–DNA adducts (*P*=0.02516), drug metabolism–cytochrome P450 (*P*=0.026244), platinum drug resistance (*P*=0.026605), metabolism of xenobiotics by cytochrome P450 (*P*=0.02841), and drug metabolism–other enzymes (*P*=0.029131) (Table 6 and Figure 10(b)).

To understand the interaction network of these genes, we used the GeneMANIA database for analysis. The results showed that these genes were in a complex PPI network, with physical Interactions of 77.64%, coexpression of 8.01%, predicted of 5.37%, colocalization of 3.63%, genetic Interactions of 2.87%, pathway of 1.88% and shared protein domains of 0.60% (Figure 10(c)). GO enrichment analysis of these 25 coexpressed genes found that, for biological processes, they were mainly enriched in the glutathione metabolic process (*P*=2.27*E* − 10), benzene-containing compound metabolic process (*P*=4.18*E* − 08), and cellular modified amino acid metabolic process (*P*=1.40*E* − 07); in terms of cell composition, it is mainly located in the intercellular bridge (*P*=2.36*E* − 06), transcription regulator complex (*P*=2.59*E* − 05), and transcription repressor complex (*P*=0.000121); and its molecular functions are mainly involved in glutathione transferase activity (*P*=1.56*E* − 10), glutathione binding (*P*=4.64*E* − 10), and oligopeptide binding (*P*=7.28*E* − 10) (Table 7 and Figure 10(d)). KEGG enrichment analysis showed that these genes were mainly enriched in the following pathways: cellular senescence (*P*=3.38*E* − 08), glutathione metabolism (*P*=1.50*E* − 07), chemical carcinogenesis-DNA adducts (*P*=1.65*E* − 05), drug metabolism–cytochrome P450 (*P*=1.95*E* − 05), platinum drug resistance (*P*=2.06*E* − 05), metabolism of xenobiotics by cytochrome P450 (*P*=2.68*E* − 05), drug metabolism-other enzymes (*P*=2.96*E* − 05), and tyrosine metabolism (*P*=7.27*E* − 05) (Table 8 and Figure 10(e)).

## 4. Discussion

At present, many studies have found many genes that affect HCC, but the mechanism of HCC occurrence and development is still not very clear, and there is an urgent need to further explore the factors affecting its development and prognosis. Although several previous studies have analyzed gene signatures related to HCC prognosis [30, 37–39], these studies did not further screen genes that are more closely related to patient survival after screening DEGs. Therefore, in this study, we used the random forest and limma algorithms to screen out 30 important genes and 1,564 DEGs, respectively, and then took the intersection of the two to further screen out 7 important DEGs: MAFG-DT, GSTZ1, GPRIN1, MYBL2, LINC00907, CCN1, and GSTM5. Subsequent enrichment analysis, expression profiling analysis, survival analysis, and the constructed prognostic prediction model indicated that they are closely related to the occurrence and prognosis of HCC.

Among the seven important DEGs, MAFG-DT (logFC = 2.295817), GPRIN1 (logFC = 2.444281), and MYBL2 (logFC = 3.861042) were all significantly elevated in liver cancer samples (Figure 2(d)). MAFG-DT is an oncogenic long noncoding RNA (lncRNA), and many previous studies have shown that overexpression of MAFG-DT can promote the proliferation and metastasis of cancer cells [40–43]. High expression of MAFG-DT is significantly associated with poor prognosis in bladder and breast cancer patients [44, 45]. In this study, MAFG-DT was highly expressed in liver cancer patients, and the normal group was significantly different from the liver cancer group after grouping by age, gender, and stage (Figures 3–6). In addition, after grouping according to the high and low expression of MAFG-DT, the survival time of the low expression group was higher than that of the high expression group, although there was no significant difference (*P*=0.19) (Figure 7). As a member of the GPRIN family, GPRIN1 acts on the classical G protein-coupled receptor pathway [46]. GPRIN1 is closely related to cancer, and it can promote the proliferation and metastasis of lung cancer by promoting the epithelial-mesenchymal transition of lung cancer cells [47]. But interestingly, Zhou et al. found that GPRIN1 is downregulated in gastric cancer cells and tissues, and it can inhibit the invasion and metastasis of gastric cancer [48]. Our results showed that GPRIN1 was highly expressed in liver cancer tissues, and patients with high GPRIN1 expression had a worse prognosis (*P*=0.002) (Figure 7). Therefore, we speculate that due to the different molecular biological mechanisms of different cancers, GPRIN1 promotes the malignant behavior of tumors in lung cancer and liver cancer but plays an inhibitory role in gastric cancer. MYBL2, a transcription factor in the myeloblastosis family, plays a key role in cell proliferation, differentiation, and cell cycle. Previous studies have shown that MYBL2 is highly expressed in cancers such as ovarian cancer and breast cancer and affects the prognosis of patients [49, 50]. Our study on liver cancer also proved that MYBL2 is highly expressed in cancer tissues, and the high-expression group has a poor prognosis (*P*=0.00047) (Figure 7).

In addition, LINC00907 (logFC = −2.6057), CCN1 (logFC = −2.05925), GSTZ1 (logFC = −2.34197), and GSTM5 (logFC = −2.8954) were lowly expressed in liver cancer tissues. Through survival analysis, only GSTM5 was found to be associated with patient prognosis (*P*=0.01) (Figure 7). The glutathione S-transferase (GST) gene family plays an important role in detoxification in the body, protecting cells from damage by poisons and charged particles. The GST family is closely related to the occurrence and development of many tumors, and the same GSTM5 as a member is abnormally expressed in ovarian cancer and nonsmall cell lung cancer [51, 52]. In this study, the expression of GSTM5 was decreased in HCC tissues and suggested a poor prognosis, which was also consistent with the previous findings, further indicating that GSTM5 plays a key role in tumorigenesis.

Next, we constructed a risk-scoring model based on the multivariate Cox regression analysis of 7 important DEGs. Kaplan‒Meier survival analysis showed that the high-risk group had a significantly lower survival time (*P* = 0.00017) (Figure 8(b)). However, subsequent ROC analysis showed that the risk-scoring model was not dominant compared to the stage (AUC = 0.659 vs 0.582) (Figure 9). However, in the ROC analysis, the AUC of the risk model is second only to the stage. Combined with the results of the Kaplan‒Meier survival analysis, we believe that the risk model still has a certain significance. Especially when the patient is in the early stage of the disease, and the stage cannot indicate the disease progression, early individualized treatment according to the risk score has extremely important value for improving the prognosis of patients.

To further study the molecular signaling pathways of important DEGs, we performed enrichment analysis and coexpression analysis. As we discussed before, both GSTZ1 and GSTM5 belong to the GST family, so the screened important DEGs were mainly enriched in the glutathione metabolic process and glutathione transferase activity (*P* = 0.00011692; *P* = 1.91*E* − 05) (Table 5), and further enrichment analysis of their interacting proteins showed the same results (*P* = 2.27*E* − 10; *P* = 1.56*E* − 10) (Table 7). The KEGG enrichment analysis showed that glutathione metabolism (*P* = 1.50*E* − 07) was still a major enrichment pathway, and interestingly, we found that more genes were enriched in cellular senescence (*P* = 3.38*E* − 08) (Table 8). In addition to the central gene MYBL2, the cellular senescence pathway has its associated genes: LIN9, LIN37, LIN54, E2F4, RBL1, and FOXM1, which together constitute the DREAM complex and play an important role in cell cycle regulation [53]. This proves that MYBL2 can also promote the progression of HCC by interfering with cell cycle regulation.

However, this study still has some limitations. Firstly, the data for model construction and validation were obtained from the TCGA database. This public database contains incomplete information, and the data are all retrospective. Therefore, prospective real-world studies are necessary to verify the reliability of the model. It should be noted that in the process of research, it is necessary to comprehensively collect data at all stages of disease progression, such as blood samples and imaging data, etc., to eliminate information distortion caused by incomplete data collection as far as possible. To improve the representativeness of the results, multicenter studies are also necessary. Secondly, the data used in this study were only from the TCGA database, which may make the results lack representative. Therefore, data from other databases, such as GEO and Oncomine databases, can be selected for subsequent analysis and cross-validation. Thirdly, the diagnostic efficacy of the risk score model constructed in this study was not superior to staging, although the results of its KM analysis were beneficial. One of the reasons for this may be due to the bias caused by the data analysis using only the TCGA database. However, compared with the stage, the risk scoring model has more important significance in the evaluation of patients in the early stage of the disease to improve the prognosis. The performance of this model will be tested in clinical cohorts in the future. Finally, the seven important DEGs screened in this study are currently only data demonstrations. We will carry out in vitro and in vivo experiments to further explore the specific molecular pathways of these genes in HCC.

## 5. Conclusion

In conclusion, we innovatively used a combination of random forest and Limma to screen out the important DEGs for HCC development. Expression analysis and survival analysis were performed, indicating that these genes are closely related to the survival of HCC patients. The subsequently constructed prognostic risk scoring model has good predictive value for the prognosis of HCC patients, and combining it with other clinical indicators can provide an effective reference for clinical treatment. Subsequent enrichment analysis and coexpression analysis showed that seven important DEGs were closely related to cellular senescence and glutathione metabolism, which also provided new ideas for further research on the molecular mechanism of HCC occurrence and development. In brief, the early risk score model provided in this study can be used as a reference for subsequent personalized treatment of patients and ultimately help to improve prognosis.

## Figures and Tables

**Table 1:** 30 important genes screened by random forest.

Ensemble	Good	Poor	Mean decrease accuracy	Mean decrease Gini
ENSG00000261461.1	3.5035258	3.4857582	3.7528651	0.508084
ENSG00000171819.5	3.5092202	2.720329	3.4323096	0.5052108
ENSG00000276984.1	2.2262274	3.0303442	3.0226219	0.4598939
ENSG00000280160.1	0.2249288	2.1851122	1.5534425	0.4370629
ENSG00000272732.1	2.7193541	2.1123561	2.9543775	0.428348
ENSG00000205955.4	1.6407665	1.4453403	1.9828496	0.421363
ENSG00000269930.1	1.678516	3.1569168	2.6375549	0.4010261
ENSG00000265688.2	0.1760152	1.291468	1.4563289	0.3938647
ENSG00000184811.4	3.1610986	3.5654376	3.5533163	0.3897576
ENSG00000135912.11	2.5015435	0.0774343	1.4520492	0.3676444
ENSG00000223656.1	1.872504	1.3207259	1.7311298	0.3409327
ENSG00000095383.20	2.9219665	2.4899488	2.8241699	0.3226696
ENSG00000100577.19	−1.168253	1.3519506	0.5418066	0.3008067
ENSG00000147588.7	1.8170996	2.4736802	2.6879344	0.2993155
ENSG00000128165.9	3.1080429	3.1663719	3.3007222	0.2966643
ENSG00000169258.7	1.5831498	2.5090588	2.4754708	0.2964592
ENSG00000101057.16	2.2969796	2.7405219	2.6271281	0.2817687
ENSG00000215386.13	1.3803304	0.0963518	0.6424683	0.2776084
ENSG00000231982.1	2.3729127	2.6866166	2.5394458	0.2699614
ENSG00000267586.7	2.1017253	−0.0713384	1.2067781	0.2620233
ENSG00000168490.14	0.3380425	1.1292257	1.0463427	0.2565421
ENSG00000142871.18	−0.4686499	0.9268551	0.3333044	0.2423563
ENSG00000275494.1	0.2773768	1.0829379	0.7525331	0.2374018
ENSG00000227959.1	1.5318984	1.7262925	1.6557142	0.2360688
ENSG00000213694.6	2.2435296	2.2237149	2.2319256	0.2287172
ENSG00000275265.1	0.3430476	0.4610777	0.6555662	0.2262146
ENSG00000253105.6	2.2140516	2.2472489	2.5262515	0.2159858
ENSG00000176485.12	0.3947449	1.7110925	1.6244757	0.2140441
ENSG00000245067.7	2.3810183	0.1541471	1.4993123	0.2139093
ENSG00000245322.7	2.4175229	−0.7897455	0.6036063	0.2138007

**Table 2:** 7 important DEGs related to HCC.

Gene symbol	LogFC	AveExpr	*t*	*P* value	Adj. *P* value	*B*	Change
MAFG-DT	2.295816916	0.180864209	8.733112195	5.68*E* − 17	9.15*E* − 16	27.82424792	Up
GPRIN1	2.444281099	0.984424466	8.88620807	1.79*E* − 17	3.09*E* − 16	28.94197713	Up
MYBL2	3.86104155	2.858320008	8.757496695	4.73*E* − 17	7.71*E* − 16	27.95856037	Up
LINC00907	−2.60570328	−3.393211756	−9.088003866	3.82*E* − 18	7.25*E* − 17	30.46891163	Down
GSTZ1	−2.341972944	4.83864018	−13.29705978	5.36*E* − 34	5.72*E* − 32	66.16025292	Down
CCN1	−2.059250199	5.163337155	−11.41610643	1.60*E* − 26	7.98*E* − 25	49.05138576	Down
GSTM5	−2.895398904	−1.505519136	−9.292124572	7.84*E* − 19	1.63*E* − 17	31.95202187	Down

**Table 3:** Multivariate Cox regression analysis of important DEGs.

Important genes	Coeff	HR	95% CI		*P*
			Lower	Upper	
MAFG-DT	0.069645	1.0721	0.8671	1.326	0.52014
GSTZ1	−0.070909	0.9315	0.4820	1.800	0.83293
GPRIN1	0.009885	1.0099	0.7659	1.332	0.94415
MYBL2	0.461418	1.5863	1.1213	2.244	0.00915
LINC00907	0.010721	1.0108	0.8732	1.170	0.88581
CCN1	0.227363	1.2553	0.6951	2.267	0.45090
GSTM5	−0.116514	0.8900	0.7918	1.000	0.05078

**Table 4:** Univariate and multivariate Cox regression analyses on the survival of HCC patients.

Variable	Univariate analysis		Multivariate analysis	
	HR (95% CI)	*P*	HR (95% CI)	*P*
Age	1.009 (0.9946–1.023)	0.23	1.0078 (0.9928–1.0229)	0.31007
Gender	0.7857 (0.5469–1.129)	0.192	0.9014 (0.6043–1.3447)	0.61101
Stage				
Stage II	1.452 (0.8705–2.423)	0.15300	1.3944 (0.8344–2.3303)	0.20446
Stage III	3.062 (1.9841–4.726)	4.31*e* − 07^^*∗∗∗*^^	2.8765 (1.8589–4.4513)	2.11*e* − 06^^*∗∗∗*^^
Stage IV	6.415 (1.9691–20.897)	0.00204^^*∗∗*^^	5.5058 (1.6529–18.3403)	0.00546^^*∗∗*^^
Risk group	0.5066 (0.353–0.727)	0.000224^^*∗∗∗*^^	0.5549 (0.3767–0.8173)	0.00287^^*∗∗*^^

HR, hazard ratio; ^^*∗∗*^^*P*  <  0.01, ^^*∗∗∗*^^*P*  <  0.001.

**Table 5:** GO enrichment results of important DEGs.

Category	ID	Description	Bg ratio	*P*value	*P*adjust	*q*value	geneID	Count
BP	GO:0006749	Glutathione metabolic process	65/18800	0.00011692	0.016368738	0.005907515	GSTZ1/GSTM5	2
MF	GO:0004364	Glutathione transferase activity	26/18410	1.91*E* − 05	0.000363455	8.05*E* − 05	GSTZ1/GSTM5	2
MF	GO:0016765	Transferase activity, transferring alkyl or aryl (other than methyl) groups	59/18410	0.000100347	0.000953296	0.000211257	GSTZ1/GSTM5	2
MF	GO:0004602	Glutathione peroxidase activity	22/18410	0.005961396	0.035906466	0.007957112	GSTZ1	1
MF	GO:0005520	Insulin-like growth factor binding	29/18410	0.00785223	0.035906466	0.007957112	CCN1	1
MF	GO:0016859	Cis-trans isomerase activity	41/18410	0.011086965	0.035906466	0.007957112	GSTZ1	1
MF	GO:0004601	Peroxidase activity	52/18410	0.014044721	0.035906466	0.007957112	GSTZ1	1
MF	GO:0050840	Extracellular matrix binding	55/18410	0.014850152	0.035906466	0.007957112	CCN1	1
MF	GO:0016684	Oxidoreductase activity, acting on peroxide as acceptor	56/18410	0.015118512	0.035906466	0.007957112	GSTZ1	1
MF	GO:0016209	Antioxidant activity	85/18410	0.022875552	0.044982892	0.009968508	GSTZ1	1
MF	GO:0051219	Phosphoprotein binding	88/18410	0.023675206	0.044982892	0.009968508	GPRIN1	1

**Table 6:** KEGG enrichment results of important DEGs.

ID	Description	Bg ratio	*P* value	*P* adjust	*q* value	geneID	Count
hsa00350	Tyrosine metabolism	36/8159	0.013180205	0.049939588	0.021903328	2954	1
hsa00480	Glutathione metabolism	57/8159	0.020814906	0.049939588	0.021903328	2949	1
hsa05204	Chemical carcinogenesis–DNA adducts	69/8159	0.02515986	0.049939588	0.021903328	2949	1
hsa00982	Drug metabolism–cytochrome P450	72/8159	0.026244087	0.049939588	0.021903328	2949	1
hsa01524	Platinum drug resistance	73/8159	0.026605317	0.049939588	0.021903328	2949	1
hsa00980	Metabolism of xenobiotics by cytochrome P450	78/8159	0.028410127	0.049939588	0.021903328	2949	1
hsa00983	Drug metabolism–other enzymes	80/8159	0.029131427	0.049939588	0.021903328	2949	1

DEGs, differentially expressed genes.

**Table 7:** GO enrichment results of interacting genes.

Category	ID	Description	Bg ratio	*P*value	*P*adjust	*q*value	geneID	Count
BP	GO:0006749	Glutathione metabolic process	65/18800	2.27*E* − 10	1.34*E* − 07	1.06*E* − 07	GSTZ1/GSTM5/GSS/GSTM4/GSTM2/GSTM1	6
BP	GO:0042537	Benzene-containing compound metabolic process	27/18800	4.18*E* − 08	1.23*E* − 05	9.79*E* − 06	FAH/GSTM4/GSTM2/GSTM1	4
BP	GO:0006575	Cellular-modified amino acid metabolic process	188/18800	1.40*E* − 07	2.74*E* − 05	2.18*E* − 05	GSTZ1/GSTM5/GSS/GSTM4/GSTM2/GSTM1	6
BP	GO:0006570	Tyrosine metabolic process	15/18800	9.35*E* − 07	0.000135472	0.000107739	GSTZ1/FAH/HGD	3
BP	GO:0009074	Aromatic amino acid family catabolic process	16/18800	1.15*E* − 06	0.000135472	0.000107739	GSTZ1/FAH/HGD	3
BP	GO:0006790	Sulfur compound metabolic process	339/18800	4.36*E* − 06	0.000394304	0.000313583	GSTZ1/GSTM5/GSS/GSTM4/GSTM2/GSTM1	6
BP	GO:0042178	Xenobiotic catabolic process	25/18800	4.69*E* − 06	0.000394304	0.000313583	GSTM4/GSTM2/GSTM1	3
BP	GO:0042759	Long-chain fatty acid biosynthetic process	29/18800	7.42*E* − 06	0.000546204	0.000434386	GSTM4/GSTM2/GSTM1	3
BP	GO:0009072	Aromatic amino acid family metabolic process	31/18800	9.11*E* − 06	0.000596212	0.000474157	GSTZ1/FAH/HGD	3
BP	GO:1901606	Alpha-amino acid catabolic process	94/18800	0.000257064	0.015141082	0.012041429	GSTZ1/FAH/HGD	3
BP	GO:1900221	Regulation of amyloid-beta clearance	20/18800	0.00031786	0.017019984	0.013535686	CLU/HDAC1	2
BP	GO:0006805	Xenobiotic metabolic process	108/18800	0.000386746	0.018101332	0.014395662	GSTM4/GSTM2/GSTM1	3
BP	GO:0001676	Long-chain fatty acid metabolic process	111/18800	0.000419095	0.018101332	0.014395662	GSTM4/GSTM2/GSTM1	3
BP	GO:0009063	Cellular amino acid catabolic process	112/18800	0.000430252	0.018101332	0.014395662	GSTZ1/FAH/HGD	3
BP	GO:1990748	Cellular detoxification	115/18800	0.000464867	0.018253777	0.014516899	GSTZ1/GSTM2/GSTM1	3
BP	GO:0006520	Cellular amino acid metabolic process	285/18800	0.000508838	0.018731601	0.014896904	GSTZ1/FAH/HGD/GSS	4
BP	GO:0097237	Cellular response to toxic substance	123/18800	0.000565787	0.019602849	0.015589791	GSTZ1/GSTM2/GSTM1	3
BP	GO:0098754	Detoxification	150/18800	0.001006626	0.032939028	0.026195814	GSTZ1/GSTM2/GSTM1	3
BP	GO:0097242	Amyloid-beta clearance	39/18800	0.001220616	0.037009268	0.029432802	CLU/HDAC1	2
BP	GO:0006633	Fatty acid biosynthetic process	162/18800	0.001256681	0.037009268	0.029432802	GSTM4/GSTM2/GSTM1	3
BP	GO:0071466	Cellular response to xenobiotic stimulus	168/18800	0.001395135	0.039130226	0.031119561	GSTM4/GSTM2/GSTM1	3
CC	GO:0045171	Intercellular bridge	75/19594	2.36*E* − 06	0.00014373	0.000109131	GSTM5/GSTM4/GSTM2/GSTM1	4
CC	GO:0005667	Transcription regulator complex	483/19594	2.59*E* − 05	0.000788585	0.000598753	LIN9/TFDP2/E2F4/LIN37/RBL1/HDAC1	6
CC	GO:0017053	Transcription repressor complex	76/19594	0.000121293	0.002466282	0.001872587	LIN9/LIN37/HDAC1	3
MF	GO:0004364	Glutathione transferase activity	26/18410	1.56*E* − 10	1.25*E* − 08	7.22*E* − 09	GSTZ1/GSTM5/GSTM4/GSTM2/GSTM1	5
MF	GO:0043295	Glutathione binding	10/18410	4.64*E* − 10	1.86*E* − 08	1.07*E* − 08	GSS/GSTM4/GSTM2/GSTM1	4
MF	GO:1900750	Oligopeptide binding	11/18410	7.28*E* − 10	1.94*E* − 08	1.12*E* − 08	GSS/GSTM4/GSTM2/GSTM1	4
MF	GO:0016765	Transferase activity, transferring alkyl or aryl (other than methyl) groups	59/18410	1.15*E* − 08	2.31*E* − 07	1.34*E* − 07	GSTZ1/GSTM5/GSTM4/GSTM2/GSTM1	5
MF	GO:0072341	Modified amino acid binding	93/18410	6.00*E* − 06	9.60*E* − 05	5.56*E* − 05	GSS/GSTM4/GSTM2/GSTM1	4
MF	GO:1901681	Sulfur compound binding	267/18410	2.10*E* − 05	0.000279468	0.000161797	CCN1/GSS/GSTM4/GSTM2/GSTM1	5
MF	GO:0042277	Peptide binding	322/18410	5.13*E* − 05	0.000586451	0.000339524	GSS/GSTM4/GSTM2/CLU/GSTM1	5
MF	GO:1990841	Promoter-specific chromatin binding	62/18410	7.00*E* − 05	0.000699951	0.000405235	E2F4/RBL1/HDAC1	3
MF	GO:0033218	Amide binding	402/18410	0.000146108	0.001298736	0.0007519	GSS/GSTM4/GSTM2/CLU/GSTM1	5
MF	GO:0004602	Glutathione peroxidase activity	22/18410	0.000370295	0.002962359	0.00171505	GSTZ1/GSTM2	2
MF	GO:0005178	Integrin binding	156/18410	0.001059937	0.007708636	0.004462895	CCN1/CIB2/ITGB5	3
MF	GO:0004601	Peroxidase activity	52/18410	0.002075466	0.01383644	0.008010571	GSTZ1/GSTM2	2
MF	GO:0016684	Oxidoreductase activity, acting on peroxide as acceptor	56/18410	0.002402771	0.014786285	0.008560481	GSTZ1/GSTM2	2
MF	GO:0016209	Antioxidant activity	85/18410	0.005443299	0.031104563	0.018007905	GSTZ1/GSTM2	2

BP, biological process; CC, cell component; MF, molecular function.

**Table 8:** KEGG enrichment results of interacting genes.

ID	Description	Bg ratio	*P* value	*P* adjust	*q* value	geneID	Count
hsa04218	Cellular senescence	156/8164	3.38*E* − 08	1.35*E* − 06	8.89*E* − 07	4605/132660/286826/2305/1874/55957/5933	7
hsa00480	Glutathione metabolism	57/8164	1.50*E* − 07	2.99*E* − 06	1.97*E* − 06	2949/2937/2948/2946/2944	5
hsa05204	Chemical carcinogenesis–DNA adducts	69/8164	1.65*E* − 05	0.000164828	0.00010844	2949/2948/2946/2944	4
hsa00982	Drug metabolism–cytochrome P450	72/8164	1.95*E* − 05	0.000164828	0.00010844	2949/2948/2946/2944	4
hsa01524	Platinum drug resistance	73/8164	2.06*E* − 05	0.000164828	0.00010844	2949/2948/2946/2944	4
hsa00980	Metabolism of xenobiotics by cytochrome P450	78/8164	2.68*E* − 05	0.00016932	0.000111395	2949/2948/2946/2944	4
hsa00983	Drug metabolism–other enzymes	80/8164	2.96*E* − 05	0.00016932	0.000111395	2949/2948/2946/2944	4
hsa00350	Tyrosine metabolism	36/8164	7.27*E* − 05	0.000363501	0.000239145	2954/2184/3081	3
hsa04110	Cell cycle	126/8164	0.000175201	0.000778672	0.000512284	7029/1874/5933/3065	4
hsa05418	Fluid shear stress and atherosclerosis	139/8164	0.000255701	0.001022805	0.000672898	2949/2948/2946/2944	4
hsa05225	Hepatocellular carcinoma	168/8164	0.000526692	0.001915242	0.001260028	2949/2948/2946/2944	4
hsa05207	Chemical carcinogenesis–receptor activation	212/8164	0.001260675	0.004202252	0.002764639	2949/2948/2946/2944	4
hsa05208	Chemical carcinogenesis–reactive oxygen species	223/8164	0.001520568	0.00467867	0.003078073	2949/2948/2946/2944	4

## Data Availability

The data used to support the findings of this study are included within the article.

## Abbreviations

CTLA4: Cytotoxic T-lymphocyte antigen 4
DEGs: Differentially expressed genes
GO: Gene ontology
HCC: Hepatocellular carcinoma
ICIs: Immune checkpoint inhibitors
KEGG: Kyoto Encyclopedia of Genes and Genomes
LAG-3: Lymphocyte activating gene 3 protein
OS: Overall survival
PD-1: Programmed death-1
PD-L1: Programmed death ligand-1
ROC: Receiver operating characteristic
TCGA: The Cancer Genome Atlas
TIM-3: Mucin domain molecule 3
VEGF: Vascular endothelial growth factor.
